# CDO1 as a prognostic biomarker and therapeutic target in gastric cancer: Mechanistic insights into the PI3K/AKT‐THBS1 axis and epigenetic reactivation by decitabine

**DOI:** 10.1002/ctm2.70784

**Published:** 2026-08-30

**Authors:** Fazhi Wang, Runkai Zhou, Jinfeng Cai, Jiazhe Wen, Abudushalamu Yalikun, Yang Yu, Yugang Wen

**Affiliations:** ^1^ Department of General Surgery, Shanghai General Hospital Shanghai Jiao Tong University School of Medicine Shanghai China; ^2^ School of Basic Medical Sciences Fudan University Shanghai China

**Keywords:** CDO1, decitabine, gastric cancer, PI3K/AKT, THBS1, tumour suppressor

## Abstract

**Background:**

As a pivotal metabolic enzyme, cysteine dioxygenase type 1 (CDO1) exerts tumour‐suppressive effects across diverse tumour types, and its expression is strongly correlated with clinical prognosis. However, the molecular mechanisms underlying CDO1‐mediated tumour suppression in gastric cancer (GC), its relationship with the tumour‐associated immune microenvironment, and pharmacological strategies to restore its expression remain poorly understood.

**Methods:**

CDO1 expression and prognosis were evaluated by multi‐omics and tissue microarray analyses. Tumour microenvironment and immune infiltration were analyzed using ESTIMATE and ssGSEA. Downstream pathways and interacting proteins were identified by transcriptomics, co‐immunoprecipitation, and GST pull‐down. CDO1 function was assessed by proliferation, apoptosis, and migration assays in gain‐ and loss‐of‐function models. In vivo tumorigenesis and CDO1‐dependent decitabine efficacy were evaluated by subcutaneous xenografts. Patient‐derived organoids were used to assess decitabine sensitivity and 5‐FU synergy.

**Results:**

Compared with normal controls, CDO1 expression was notably decreased in GC tissues, and its low expression was strongly linked to unfavourable prognosis, supporting its utility as a biomarker for prognosis. Elevated CDO1 levels correlated with an immune‐active tumour microenvironment and reduced metastatic signatures. Mechanistically, CDO1 directly bound to PI3K p85α, disrupting p85α‐p110α dimerization, thereby attenuating PI3K/AKT phosphorylation and downregulating THBS1 expression. CDO1 overexpression led to reduced proliferation, invasiveness, and EMT, accompanied by increased apoptosis. These effects were reversed by PI3K activation or THBS1 co‐overexpression. Decitabine was identified as an agent that epigenetically restores CDO1 expression. Critically, CDO1 knockdown significantly attenuated the anti‐tumour efficacy of decitabine in vivo, confirming that decitabine acts primarily through CDO1 reactivation. Decitabine synergized with 5‐FU in both organoids and xenografts.

**Conclusions:**

Our data identify CDO1 as both a biomarker for prognosis and a tumour suppressor in gastric cancer. They reveal a CDO1–PI3K/AKT–THBS1 signalling axis and support the epigenetic reactivation of CDO1 by decitabine as a translatable therapeutic strategy.

**Key points:**

- CDO1 is frequently downregulated in gastric cancer and serves as an independent favourable prognostic biomarker.
- CDO1 directly binds PI3K p85α, disrupting p85α–p110α dimerization to suppress the PI3K/AKT–THBS1 signalling axis.
- Decitabine epigenetically restores CDO1 expression, and its anti‐tumour activity is critically CDO1‐dependent in vivo.
- Combining decitabine with 5‐FU synergistically overcomes gastric cancer growth in patient‐derived organoids and subcutaneous xenograft models.

## Graphical abstract

CDO1 is a tumour suppressor in gastric cancer that directly binds PI3K p85α, disrupting p85α–p110α dimerization to inhibit the PI3K/AKT–THBS1 axis. Decitabine epigenetically restores CDO1 expression and exerts CDO1‐dependent anti‐tumour activity, synergizing with 5‐FU.

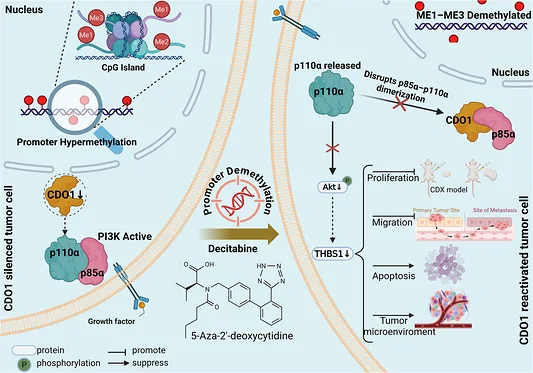

## 1 INTRODUCTION

GC continues to pose a major global health challenge, ranking as the fifth most common cancer and the fourth leading cause of cancer death worldwide, with 2022 estimates reporting .97 million new cases and .66 million fatalities.^1^ The disease is commonly detected at an advanced or metastatic stage, which, coupled with its aggressive biology and scarcity of effective therapeutic targets, underpins the persistently unfavourable prognosis.^2^ Despite advances in combined‐modality regimens, including immunotherapy and targeted agents, the average 5‐year survival rate remains below 30%.^3^ This stark reality highlights the urgent requirement for new biomarkers and actionable therapeutic targets to refine risk stratification and improve clinical outcomes in GC.^4^, ^5^

CDO1 functions as a non‐heme iron‐dependent dioxygenase that catalyzes the conversion of L‐cysteine to cysteine sulfinic acid (CSA) via irreversible oxidation, representing the first committed step in the taurine biosynthesis pathway.^6^ This reaction requires molecular oxygen and ferrous iron (Fe^2^^+^) as cofactors, and CSA is subsequently decarboxylated to hypotaurine and then oxidized to taurine. As the rate‐limiting enzyme of cysteine catabolism, CDO1 is essential for maintaining intracellular cysteine homeostasis and redox balance by diverting cysteine away from glutathione (GSH) and coenzyme A synthesis.^7^ In normal physiology, CDO1 exhibits high expression levels in the liver, kidney, brain, and adipose tissues.^8^, ^9^ Accumulating evidence has established CDO1 as a bona fide tumour suppressor in multiple malignancies, including breast, colorectal, hepatocellular, gastric, and oral squamous cell carcinomas.^10^, ^11^, ^12^, ^13^ In these cancers, CDO1 is frequently silenced by promoter CpG island hypermethylation—an epigenetic mechanism distinct from the mutational inactivation typical of classical tumour suppressors including p53 or PTEN.^14^, ^15^, ^16^ Loss of CDO1 expression leads to cysteine accumulation and enhanced GSH production, conferring resistance to oxidative stress and ferroptosis.^17^, ^18^, ^19^ More recently, CDO1 has been implicated in remodelling the tumour immune microenvironment through cysteine depletion and taurine production, thereby affecting both tumour cell proliferation and immune cell function.^6^, ^20^, ^21^, ^22^ Despite these insights, the downstream signalling pathways through which CDO1 executes tumour‐suppressive functions in GC, and whether its expression can be pharmacologically restored for therapeutic benefit, remain poorly understood.

Given that CDO1 silencing in GC is driven by promoter hypermethylation—a pharmacologically reversible epigenetic alteration—we reasoned that restoring CDO1 expression could represent a viable therapeutic strategy. Intriguingly, emerging evidence indicates that metabolic enzymes can exert non‐canonical tumour‐suppressive functions through direct protein–protein interactions, independent of their catalytic activity.^23^, ^24^, ^25^ The PI3K/AKT signalling is a master regulator governing cell proliferation and EMT in GC, and its hyperactivation is closely linked to aggressive disease and poor prognosis.^4^ We therefore hypothesized that CDO1 may function beyond its metabolic role—it may physically interact with and inhibit key regulatory subunits of the PI3K/AKT signalling module to suppress gastric cancer progression.

If CDO1 directly antagonizes PI3K/AKT signalling, then restoring its expression in CDO1‐silenced tumours should suppress this oncogenic pathway. The DNA methyltransferase (DNMT) inhibitor decitabine is a clinically approved agent that reverses promoter hypermethylation and restores expression of epigenetically silenced tumour suppressor genes.^26^ We therefore posited that decitabine could serve as a targeted strategy to epigenetically reactivate CDO1, thereby disrupting PI3K/AKT‐driven oncogenic signalling. However, whether decitabine‐mediated CDO1 reactivation exerts CDO1‐dependent anti‐tumour activity in vivo, and whether this approach can synergize with standard chemotherapy, remains to be determined.

Here, we identify CDO1 as a tumour‐suppressive factor in GC that operates through a CDO1–PI3K/AKT–THBS1 signalling axis. Our data reveal that CDO1 is commonly downregulated in GC and that its reduced expression independently predicts poor prognosis. Mechanistically, CDO1 directly binds the PI3K regulatory subunit p85α, disrupting p85α–p110α heterodimerization to suppress AKT phosphorylation and THBS1 expression, thereby inhibiting tumour cell proliferation, migration, and EMT. Crucially, we show that decitabine epigenetically restores CDO1 expression, and its in vivo anti‐tumour efficacy is critically dependent on CDO1 reactivation, as CDO1 knockdown largely abolishes decitabine‐mediated tumour suppression. Therapeutically, decitabine synergizes with 5‐FU in patient‐derived organoids and xenograft models. Together, these observations establish CDO1 as both a prognostic biomarker and a therapeutic target in gastric cancer, and support epigenetic reactivation of CDO1 as a translatable therapeutic strategy.

## 2 METHODS

### 2.1 CDO1 expression in clinical samples

Gastric cancer and matched adjacent non‐tumour tissues were obtained from 30 patients who underwent surgical resection at Shanghai General Hospital. Written informed consent was secured from each participant, and ethical approval was granted by the institutional review board (approval number: 2024SQ258).

### 2.2 Data acquisition and processing

Clinical and transcriptomic data for stomach adenocarcinoma (STAD) were obtained from The Cancer Genome Atlas (TCGA), with corresponding healthy gastric tissue RNA‐seq data sourced from the Genotype‐Tissue Expression (GTEx) portal. Additional gene expression profiles, including GSE183904 and GSE66229, were downloaded from the Gene Expression Omnibus (GEO). Epithelial–mesenchymal transition (EMT)‐related gene sets were derived from the EMTome resource.^27^ For immune characterization, an 18‐gene T‐cell inflammation signature and a panel of 22 inhibitory immune checkpoint molecules were used.^28^ Raw RNA‐seq count data from TCGA were analyzed using the edgeR R package, and microarray datasets were normalized and analyzed using limma. DEGs were filtered using cutoffs of FDR < .05 and |log2FC| ≥ 1.

### 2.3 Transcriptome sequencing

Total RNA was isolated from control (NC) and CDO1‐overexpressing (CDO1‐OE) AGS cells using TRIzol reagent, with three biological replicates per group. Library construction and sequencing were performed by Servicebio (Wuhan, China) on the Illumina NovaSeq 6000 platform. The RNA‐seq data generated in this study are available in the Gene Expression Omnibus (GEO) under accession number GSE339180.

### 2.4 Cell culture

Cells were sourced from the General Surgery Laboratory of Shanghai General Hospital. All cells were maintained in RPMI‐1640 medium (Gibco, USA) supplemented with 10% fetal bovine serum (FBS; Gibco) and 1% penicillin–streptomycin (Beyotime, China) at 37°C in a humidified atmosphere with 5% CO_2_.

### 2.5 Cell transfection and lentivirus

For CDO1 or THBS1 overexpression, full‐length CDO1 or THBS1 cDNA was cloned into a lentiviral expression vector. For CDO1 knockdown, a short hairpin RNA (shRNA) targeting CDO1 was cloned into a lentiviral shRNA vector. Lentiviral particles were packaged in HEK293T cells and used to infect AGS, MKN‐28, or HGC‐27 cells. Stable populations were obtained by puromycin selection (30 µM, 48 h), and efficiency was verified by qRT–PCR and western blotting. For transient CDO1 silencing, three independent siRNA sequences targeting CDO1 (GenePharma, China) were transfected into cells according to the manufacturer's protocol; the most effective siRNA (si‐CD01 #2) was selected for functional assays.

### 2.6 Patient‐derived organoid culture

Gastric cancer organoids were established from surgically resected tumour tissues. Briefly, fresh tumour tissues were minced and digested with primary tissue digestion solution (JieFangKaiRui, JFKR‐ZXHY‐100) at 37°C for 1–2 h. Dissociated cells were filtered through a 50 µm cell strainer, washed with primary washing buffer (JFKR‐ZHCY‐250), and embedded in Matrigel (Corning). The Matrigel‐cell suspension was plated as domes in 24‐well plates and allowed to solidify at 37°C for 30 min. Organoids were cultured in human gastric cancer organoid medium (JFKR‐GC‐100) at 37°C with 5% CO_2_. For normal gastric organoids, human normal gastric organoid medium (JFKR‐NG‐100) was used. Medium was renewed at 3‐day intervals. Organoids were passaged every 12 days using organoid digestion solution (JFKR‐XHY‐100) for dissociation, washed with passage washing buffer (JFKR‐HCY‐250), and re‐embedded in Matrigel.

For drug sensitivity assays, organoids were dissociated into single cells using organoid digestion solution (JFKR‐XHY‐100), seeded in 96‐well plates at 2000 cells per well in 10 µL Matrigel, and cultured for 3 days before treatment. Organoids were treated with decitabine (0, 10, 100, or 500 µM) alone or in combination with 5‐fluorouracil (100 µM) for 72 h. Cell viability was determined using the CellTiter‐Glo 3D kit (Promega, G9682).

### 2.7 Functional enrichment analyses

Gene Ontology (GO), Kyoto Encyclopedia of Genes and Genomes (KEGG), and gene set enrichment analysis (GSEA) were performed using the clusterProfiler R package. Single‐sample GSEA (ssGSEA) and gene set variation analysis (GSVA) were conducted using the GSVA and GSEABase R packages.^29^

### 2.8 Tumour microenvironment analysis

Immune and stromal scores were generated using the ESTIMATE R package. Immune cell infiltration was assessed by ssGSEA and deconvolution algorithms via the TIMER2.0 database. Activity of the tumour immune cycle was evaluated using the TIP platform.^30^

### 2.9 Real‐time quantitative reverse transcription PCR (RT‐qPCR)

RNA was extracted with an RNA Simple Total RNA Kit (Vazyme) and checked on a Nanodrop (260/280 ≥ 1.8, 260/230 ≥ 2.0), reverse‐transcribed with a HiScript II First Strand cDNA Synthesis Kit (Vazyme), and quantified by SYBR Green qPCR on an ABI PRISM 7300 system. Gene expression was calculated by the 2−ΔΔCT method with normalization to β‐actin. Primer sequences are listed in Table S1.

### 2.10 Western blot analysis

For cultured cells, monolayers were washed three times with PBS and lysed in 450 µL RIPA buffer per 10 cm dish. Lysates were clarified by centrifugation at 12 000×*g* for 10 min at 4°C, and the BCA assay was used to quantify protein concentrations. Blocking was performed using protein‐free rapid blocking buffer (Servicebio, G2052) for 10 min and incubated overnight at 4°C with primary antibodies: CDO1 (Proteintech, 12589‐1‐AP, 1:1000), β‐actin (Proteintech, 20536‐1‐AP, 1:5000), GAPDH (Proteintech, 10494‐1‐AP, 1:10 000), PCNA (Proteintech, 10205‐2‐AP, 1:5000), E‐cadherin (Proteintech, 20874‐1‐AP, 1:10 000), P21 (Proteintech, 10355‐1‐AP, 1:1000), THBS1 (ABMART, TD6848, 1:1000), PI3K p110α (STARTER, S‐3130, 1:1000), PI3K p85α (STARTER, S‐328‐24, 1:1000), p‐PI3K p85α (ABSIN, abs147914, 1:1000), AKT (STARTER, S‐652‐20, 1:1000), p‐AKT (STARTER, S‐510‐64, 1:1000), and PTEN (STARTER, SDT‐R069, 1:1000). After washing, HRP‐linked secondary antibodies were applied to the membranes for 90 min. Signals were developed with enhanced chemiluminescence substrate (Beyotime, P0018AM).

### 2.11 Cell proliferation, migration, and invasion assays

Cell viability was assessed using the CCK‐8 kit (Beyotime, C0039) as per the manufacturer's instructions. For colony formation, cells were seeded in 6‐well plates, cultured for 10 days, fixed with 4% paraformaldehyde, stained with Giemsa, and quantified. EdU incorporation was detected using an EdU assay kit (Beyotime, C0085). Wound healing was assessed by introducing a scratch into confluent monolayers and measuring closure after 48 h. Transwell invasion was evaluated using Matrigel‐coated chambers, with cells seeded in serum‐free medium in the upper chamber and complete medium as a chemoattractant in the lower chamber.

### 2.12 Cell apoptosis and cycle analysis

For cell cycle analysis, tumour cells were harvested, fixed overnight in 70% ethanol at 4°C, and stained with PI/RNase staining buffer (Liankebio, CCS‐012) at 37.5°C for 20 min. For apoptosis analysis, cells were stained with an Annexin V‐APC/7‐AAD kit (Vazyme) according to the manufacturer's protocol. Both cell cycle and apoptosis samples were analyzed by flow cytometry, and data were quantified using FlowJo software.

### 2.13 TUNEL immunofluorescence

Apoptosis in xenograft tumour tissues was detected using a FITC‐conjugated TUNEL assay kit (Vazyme, A112). Nuclei were counterstained with DAPI. Images were acquired under a fluorescence microscope.

### 2.14 CDX model

For the CDO1/THBS1 overexpression xenograft experiment, AGS cells stably expressing empty vector (NC), CDO1 (CDO1‐OE), THBS1 (THBS1‐OE), or both CDO1 and THBS1 (CDO1+THBS1) were subcutaneously injected into 4‐week‐old male BALB/c nude mice (1 × 10^7^ cells/mouse, *n* = 5 per group). Tumour dimensions were measured every 4 days, and volume was calculated as *V* = (length × width^2^)/2. At the experimental endpoint, tumours were harvested, weighed, and processed for histological analysis. For the CDO1‐dependent decitabine efficacy experiment, AGS‐sh‐NC or AGS‐sh‐CD01 cells (1 × 10^7^) were injected subcutaneously into 4‐week‐old male nude mice to test CDO1‐dependent decitabine efficacy in vivo. Once tumours attained a volume of roughly 50 mm^3^, animals were randomly allocated to four groups (*n* = 5 per group): sh‐NC + vehicle, sh‐NC + decitabine (2 mg/kg, i.p., three times weekly), sh‐CD01 + vehicle, and sh‐CD01 + decitabine. Tumour growth was monitored every four days for 28 days. At the endpoint, tumours were harvested for immunohistochemical analysis of CDO1, Ki‐67, and cleaved caspase‐3.

### 2.15 Drug sensitivity analysis

The GSCA platform^31^ was employed to integrate and analyze drug sensitivity data derived from both the Genomics of Drug Sensitivity in Cancer^32^ and the Cancer Therapeutics Response Portal ^33^ databases to evaluate the correlation between CDO1 expression and drug response. The built‐in algorithm of the platform subsequently correlated the enrichment scores (ESs) of our gene set with the half‐maximal inhibitory concentration (IC_50_) values for a wide array of anticancer compounds. Drugs exhibiting a negative correlation (Pearson's *r* < −.15, *p* < .05) were considered candidates for which tumours with a high signature score might be more sensitive.

### 2.16 Immunohistochemistry

Deparaffinized and rehydrated sections underwent heat‐induced antigen retrieval in citrate buffer (pH 6.0). Sections were blocked, incubated with primary antibodies (4°C, overnight), and detected with HRP‐conjugated secondary antibodies. DAB was used as the chromogen, and nuclei were visualized with haematoxylin. CDO1 staining was quantified by H‐score (intensity: 0–3 × % positive cells). Ki‐67 and Cleaved Caspase‐3 were scored as the percentage of positive cells.

### 2.17 Co‐immunoprecipitation and GST pull‐down

Co‐IP assays were performed using a Co‐IP kit (Absin, abs955). For GST pull‐down, purified GST or GST‐CD01 fusion proteins were incubated with His‐tagged p85α protein in binding buffer for 2 h at 4°C. Glutathione–Sepharose beads were added and incubated for an additional 1 h. After washing, bound proteins were eluted and analyzed by western blotting with anti‐His antibody.

### 2.18 Molecular docking

Molecular docking between decitabine and CDO1 was performed using AutoDock Vina, and a 100 ns molecular dynamics simulation was conducted with GROMACS 2019 to assess the stability of the predicted complex.

### 2.19 Statistical analysis

Statistical analysis was conducted using GraphPad Prism 10.1 and R 4.4.2. Data represent mean ± SD (*n* ≥ 3 independent experiments). Two‐group comparisons used unpaired two‐tailed *t*‐tests; multiple groups were compared by one‐way ANOVA with Tukey's HSD. The 2 × 2 xenograft interaction was tested by two‐way ANOVA. Survival was analyzed by log‐rank test. Correlations used Spearman's method. Categorical data were compared by χ^2^ or Fisher's exact test. *p* < .05 denoted statistical significance.

## 3 RESULTS

### 3.1 CDO1 expression is downregulated in GC patients

**FIGURE 1:**
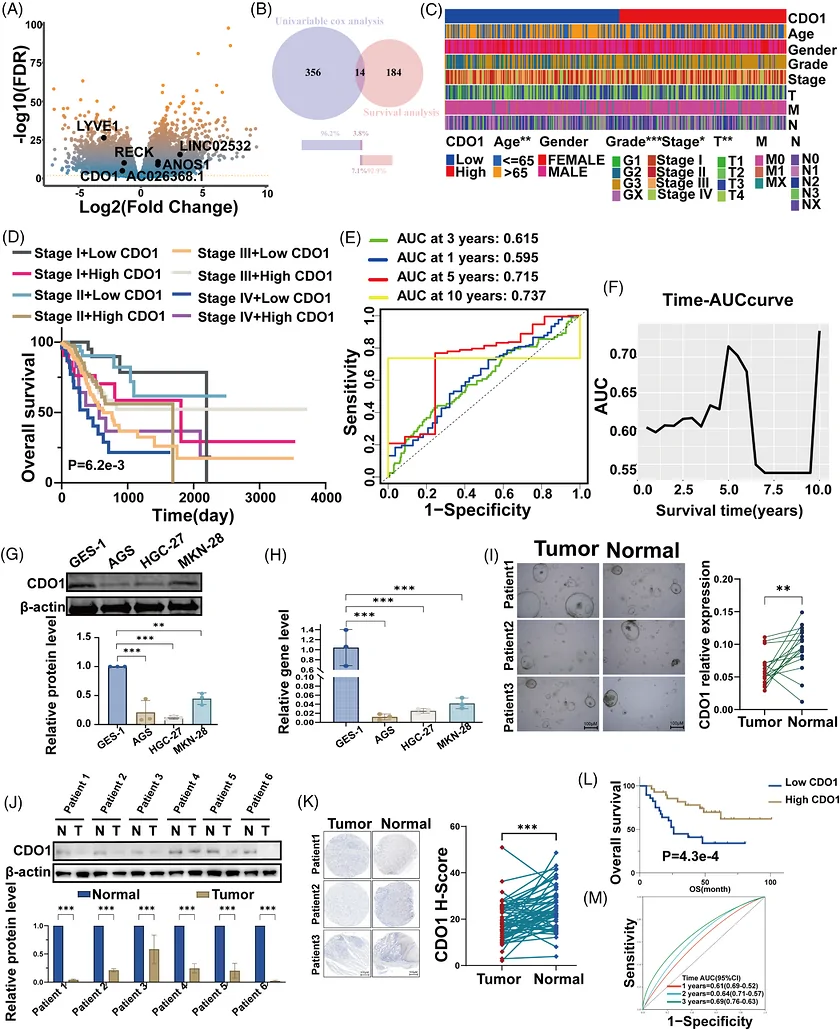
CDO1 downregulation is consistently observed across gastric and other cancers compared with normal tissues. (A) DEGs in TCGA‐STAD were identified with thresholds of *p* < .05 and |log2(fold change)| > 1. (B) Venn diagram identifying overlapping genes between univariate Cox regression and survival analyses. (C) Correlation between CDO1 expression and clinicopathological features in TCGA‐STAD. Spearman's rank correlation coefficients were calculated between CDO1 mRNA levels and each clinical variable. (D) Patients with higher CDO1 levels combined with stage III/IV disease had longer overall survival (OS) in the TCGA‐STAD cohort. (E, F) Time‐dependent ROC curve for evaluating the predictive performance of CDO1 expression for survival in patients with STAD. (G, H) Western blot and qRT‐PCR confirmation of CDO1 downregulation in various gastric cancer cell lines. (I) Representative IHC images and H‐score quantification of CDO1 in 18 paired normal and tumour gastric organoids (scale bar = 100 µm). (J) CDO1 protein is downregulated in gastric cancer tissues. CDO1 protein expression is reduced in gastric cancer tissues. (K, L) CDO1 downregulation and its prognostic significance in the TMA cohort (scale bar = 100 µm). (M) Time‐dependent ROC analysis (*p < .05, **p < .01, ***p < .001, ns = not significant. Data are presented as mean ± SD; *n* = 3.).

Analysis of the TCGA‐STAD cohort revealed 2615 differentially expressed genes (DEGs) (Figure 1A). We subsequently performed survival analysis and Cox univariate analysis separately with the criterion of *p *< .05 and screened 14 genes by taking the intersection of the results of the two methods (Figure 1B). The Correlation between candidate gene expression levels and clinicopathological parameters was then evaluated. Among the 14 genes, CDO1 showed the most significant associations across multiple clinicopathological characteristics, such as TNM stage and histological grade (Figure 1C). Based on this evidence and the supporting literature,^20^ we selected CDO1 as the tumour‐associated candidate gene. Patients with higher CDO1 expression alongside late‐stage (III/IV) gastric cancer had favourable overall survival outcomes according to TCGA‐STAD analysis (Figure 1D). For external validation of the prognostic relevance of CDO1, we analyzed two independent GEO datasets, GSE26901 and GSE66229 (Figure S1A). The prognostic performance of CDO1 was assessed by time‐dependent ROC analysis. AUC values at 1, 3, 5, and 10 years were .595, .675, .715, and .737, respectively (Figure 1E,F). These results indicate that CDO1 has greater predictive power for long‐term outcomes than for short‐term outcomes. Additional validation across the GSE13861 and GSE28541 datasets supported these AUC findings (Figure S1B). Gastric cancer tissues consistently exhibited lower CDO1 expression than normal controls, as confirmed by paired and unpaired differential analyses (Figure S1C,D). This reduced expression pattern extended to the pan‐cancer level across the UCSC XENA and GENT2 repositories (Figure S1E,F). For experimental confirmation, we assessed CDO1 expression in a panel of cell lines by qRT‒PCR and western blotting, both confirming its marked downregulation relative to GES‐1 cells (Figure 1G,H). Similarly, CDO1 mRNA levels were reduced in 17 paired patient‐derived organoids (Figure 1I). In clinical specimens, tumour tissues exhibited significantly decreased CDO1 expression compared with matched adjacent normal tissues, as evidenced by western blotting (*n* = 6 pairs) and immunohistochemistry on a tissue microarray (*n* = 56 pairs) (Figure 1J,K). Kaplan–Meier analysis of this TMA cohort moreover indicated that low CDO1 expression was linked to significantly shorter overall survival, and the prognostic value of CDO1 was further corroborated by time‐dependent ROC analysis. (Figure 1L,M). Collectively, these multi‐level analyses across cell lines, patient tissues, and organoids consistently demonstrate that CDO1 is significantly downregulated in gastric cancer.

### 3.2 CDO1 is an independent favourable prognostic indicator and enhances prognostic stratification in gastric cancer

We next explored the potential associations between CDO1 expression and clinicopathological characteristics in the TMA cohort (*n* = 56). As summarized in Table 1, low CDO1 expression was correlated with perineural invasion, diffuse Lauren type, and lymphovascular invasion. These findings indicate that CDO1 downregulation correlates with aggressive tumour features and disease progression in gastric cancer. Clinically, low CDO1 expression was correlated with adverse clinicopathological features, showing a significant association with the more aggressive tubular and proliferative subtypes of gastric cancer (Figure S2A,B). A meta‐analysis of survival data further established that high CDO1 expression was a consistent indicator of favourable overall survival in patients with GC (Figure S2C). Multivariate Cox regression demonstrated that high CDO1 levels were an independent predictor of favourable prognosis (HR = .59, 95% CI: .41–.83), as were patient age and tumour stage (Figure S2D). To evaluate its clinical utility, we integrated CDO1 expression with standard TNM staging in a prognostic model. This combined model revealed superior predictive ability for patient survival (AUC = .661) compared with models using either variable alone and was further validated by a lower AIC and a higher C‑index (Figure S2E,F). Based on the identified independent prognostic factors, a nomogram was established by integrating CDO1 expression, tumour stage, and age to provide a quantitative estimate of individual survival probability (Figure S2G). The predictive model demonstrated accurate calibration, as evidenced by the high consistency between the observed and predicted 1‐, 3‐, and 5‐year survival rates (Figure S2H). Taken together, these results substantiate CDO1 as an independent favourable prognostic indicator for gastric cancer and support its integration into improved prognostic stratification models.

**TABLE 1:** Clinical characteristics of gastric cancer patients stratified by CDO1 protein expression in the in‐house TMA cohort (*n* = 56).

Clinical characteristics	CDO1‐low (*n* = 28)	CDO1‐high (*n* = 28)	*p*‐value
Age			.686
≤65	25 (89.3%)	24 (85.7%)	
>65	3 (10.7%)	4 (14.3%)	
Gender			.736
Male	22 (78.6%)	23 (82.1%)	
Female	6 (21.4%)	5 (17.9%)	
Grade			.716
G1–G2	8 (28.6%)	11 (39.3%)	
G3–G4	20 (71.4%)	17 (60.7%)	
TNM stage			.162
Stage I–II	3 (10.7%)	7 (25%)	
Stage III–IV	25 (89.3%)	21 (75%)	
T stage			.537
T1‐2	6 (21.4%)	8 (28.6%)	
T3‐4	22 (78.6%)	20 (71.4%)	
M stage			.225
M0	5 (17.9%)	2 (7.1%)	
M1	23 (82.1%)	26 (92.9%)	
N stage			.387
N0	4 (14.3%)	2 (7.1%)	
N1	24 (85.7%)	26 (92.9%)	
Lymphovascular invasion			.041
Yes	12 (42.9%)	5 (17.9%)	
No	16 (57.1%)	23 (82.1%)	
Perineural invasion			.001
Yes	21 (75%)	6 (21.4%)	
No	7 (25%)	22 (78.6%)	
Lauren			.043^a^
Intestinal	10 (35.7%)	16 (57.1%)	
Diffuse	16 (57.1%)	9 (32.1%)	
Mixed	2 (7.1%)	3 (10.7%)	

*Note*: Data are presented as *n* (%). *p*‐values were calculated using the Chi‐squared test unless otherwise indicated.

^a^ Fisher's exact test was used when expected cell counts were <5.

### 3.3 High CDO1 expression is correlated with an enhanced immunogenic microenvironment

**FIGURE 2:**
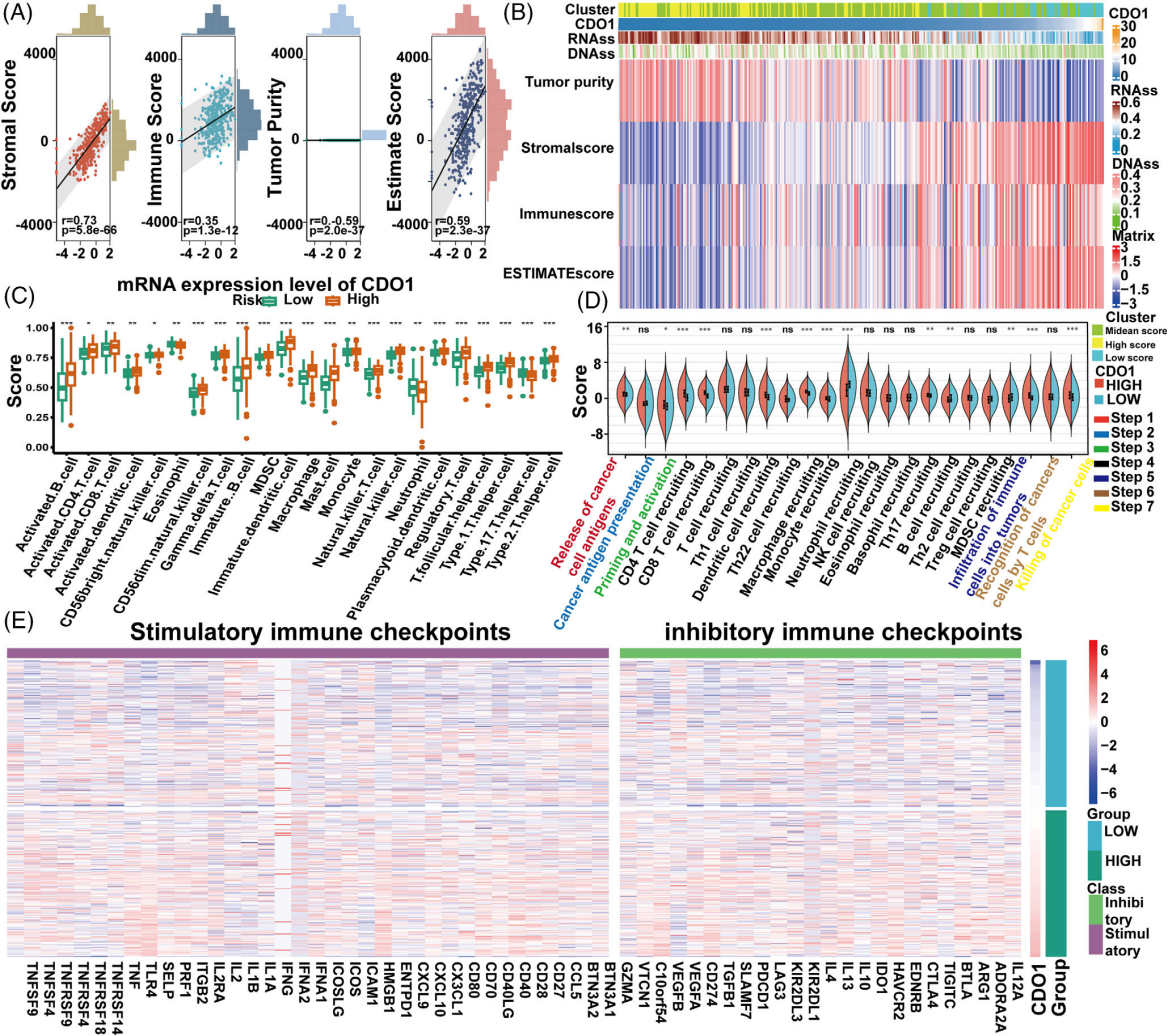
Associations between CDO1 expression and tumour metastatic capacity and tumour immunity status in gastric cancer. (A) Correlation analysis between CDO1 expression and tumour microenvironment (TME) components estimated by the ESTIMATE algorithm. (B) A summary heatmap illustrating the overall relationships among CSC scores, key TME features and CDO1 expression. (C) Differential analysis comparing the infiltration levels of tumour‐infiltrating immune cells in the CDO1‐high and CDO1‐low groups quantified by ssGSEA. (D) Activity scores of the cancer immunity cycle. (E) Correlation heatmap depicting the relationship between CDO1 expression and the transcriptional levels of key stimulatory and inhibitory immune checkpoint molecules. (*p < .05, **p < .01, ***p < .001, ns = not significant).

ESTIMATE analysis revealed that CDO1 expression correlated positively with ESTIMATE, immune, and stromal scores and negatively with tumour purity, indicating its association with a stromal‐ and immune‐enriched TME (Figure 2A). These trends are summarized in a comprehensive heatmap (Figure 2B). Further immune deconvolution analyses across seven independent algorithms consistently revealed that high CDO1 expression was linked to enhanced infiltration of monocytes, macrophages, and CD8+ T cells (Table 2), a result that was corroborated by ssGSEA (Figure 2C). Examination of the tumour immune cycle showed higher activity scores for T‐cell recruitment and infiltration in the high‐CDO1 group (Figure 2D). Furthermore, differential expression analysis of immune checkpoints revealed downregulation of TIGIT and upregulation of CD274, BTLA, and PDCD1 in the high‐CDO1 group (Figure 2E), suggesting that GC with high CDO1 expression may be more responsive to immune checkpoint inhibitors targeting CD274, BTLA, or PD‐1. To connect CDO1 with metastatic potential, we analyzed epithelial–mesenchymal transition (EMT) signatures via GSVA. CDO1 expression was significantly correlated with EMT‐related pathways in GC (Figure S3A). Moreover, cross‐cohort ssGSEA indicated that enhanced immune status was generally linked to suppressed metastatic signatures (Figure S3B–F). Together, our findings position CDO1 as a potential link between an immune‐active TME and suppressed metastasis in patients with GC.

**TABLE 2:** Correlation between CDO1 expression and tumour‐infiltrating immune cells evaluated by seven different algorithms in the TCGA‐STAD cohort.

Immune cells	TIMER	CIBERSORT	CIBERSORTABS	QUANTISEQ	MCPCOUNTER	XCELL	EPIC
B‐cell	.21	.29	.27	.37	.42	.15	0.36
CD4^+^ T‐cell	.47	−.15	.4	.21	ns	.28	0.18
CD8^+^ T‐cell	.33	.14	.37	.26	.24	.23	ns
Neutrophil	.38	−.18	−.33	Ns	.09	.10	ns
Macrophage	.64	M0: −.19	M1: .25	M1: −.12	.39	.13	0.32
		M2: .21	M2: .51	M2: .56			
Monocyte	Ns	.25	.42	.17	.39	.23	ns
NK cell	ns	−.18	−.15	ns	.37	ns	ns
Dendritic	.44	.09	.15	.17	.61	.31	ns

*Note*: Data represent Spearman's rank correlation coefficients based on the TCGA‐STAD cohort (*n* = 375). ns, not significant (*p *≥ .05). *p*‐values were adjusted for multiple testing using the Benjamini–Hochberg method. Specific coefficients are shown only when the adjusted *p* < .05.

### 3.4 Single‐cell analysis reveals an inverse association between CDO1 expression and proliferative/migratory capacity in epithelial subclusters

To complement bulk RNA analyses, we investigated the expression landscape of CDO1 at single‐cell resolution via the publicly available scRNA‐seq dataset GSE183904. Stringent quality control yielded 146 205 cells for further investigation. Unsupervised clustering and dimensionality reduction identified nine major cell lineages within the tumour microenvironment (Figure S4A). A bubble plot was performed to visualize the scaled levels of canonical lineage markers along with their cellular proportions (Figure S4B). The relative abundance of these populations varied across the samples (Figure S4C), and a volcano plot highlighted genes differentially expressed across these lineages (Figure S4D). CDO1 expression was observed in diverse stromal and immune populations, such as fibroblasts, mast cells, and T cells. Given that gastric cancer originates in the epithelial compartment, we subsequently isolated and re‐clustered 12 000 epithelial cells, resolving seven distinct subpopulations (Figure S4E). Using a gene signature derived from TCGA‐STAD differential expression analysis, each epithelial cell was assigned malignant and non‐malignant scores, leading to the classification of 8000 cells as malignant and 4000 as non‐malignant (Figure S4F). The distribution of these epithelial subpopulations differed notably between gastric cancer tissues and matched normal tissues (Figure S4G). Consistent with the bulk findings, CDO1 levels were significantly reduced in malignant cells compared with non‐malignant epithelial cells (Figure S4H). We next characterized CDO1 expression across the six epithelial subclusters by violin plot and observed that CDO1 was most abundantly expressed in cluster G1 (Figure S4I). Subsequently, we evaluated the proliferation and migration scores of each cluster using the AddModuleScore function. Notably, clusters with higher CDO1 expression, particularly G1, exhibited relatively lower proliferation and migration scores (Figure S4J,K), suggesting that CDO1 expression may be inversely associated with proliferative and migratory capacities in gastric cancer epithelial cells.

### 3.5 CDO1 exerts tumour‐suppressive effects in gastric cancer, both by curtailing proliferation via G1/S arrest and apoptosis and by attenuating metastasis through EMT inhibition

**FIGURE 3:**
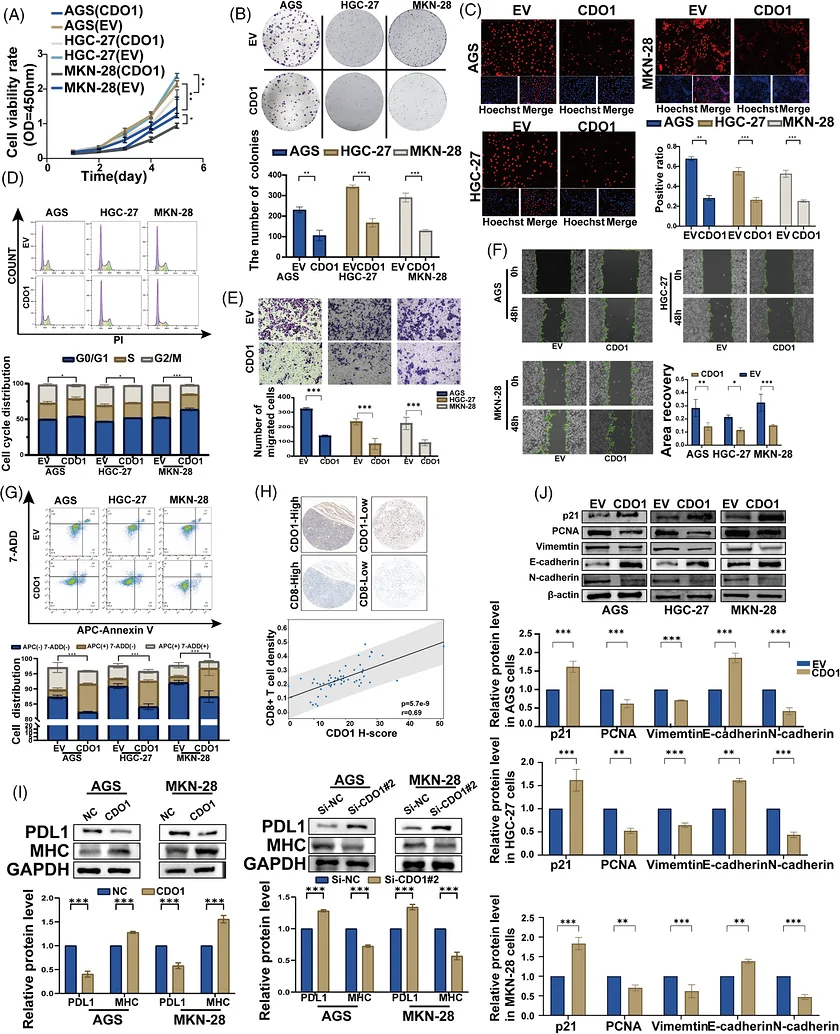
CDO1 upregulation suppressed GC cell proliferation and attenuated both migratory and invasive capacities. (A–C) Overexpression of CDO1 suppressed proliferative capacity, as demonstrated by a CCK‐8 assay, a colony formation assay, and an EdU incorporation assay (scale bar = 100 µm). (D, G) CDO1 overexpression led to cell cycle arrest and increased apoptosis in the indicated cell lines, as shown by flow cytometry. (E, F) Migration and invasion capacities were evaluated by wound‐healing and Matrigel invasion assays, respectively (scale bar = 50 µm). (H) CDO1 expression positively correlates with CD8^+^ T cell infiltration in TMA. (I) CDO1 regulates PD‐L1 and CALR expression in GC cells. (J) Western blot analysis of proliferation, EMT, and PI3K pathway marker expression following CDO1 overexpression in GC cell lines. (*p < .05, **p < .01, ***p < .001, ns = not significant. Data are presented as mean ± SD; *n* = 3).

We generated stable CDO1‐overexpressing AGS, MKN‐28, and HGC‐27 cell lines via lentiviral transduction, with overexpression efficiency confirmed by qRT–PCR and western blotting (Figure S5A,B). Functional assays revealed that CDO1 upregulation consistently and notably inhibited cell growth, as shown by the data from the CCK‐8, colony formation, and EdU incorporation assays (Figure 3A–C). Mechanistically, this growth inhibition was attributed to pronounced arrest at the G1/S phase transition (Figure 3D), accompanied by increased p21 and decreased PCNA protein levels (Figure 3J). We next examined the impact of CDO1 on metastatic behaviour. Wound healing and Transwell invasion assays showed that CDO1 overexpression inhibited the migratory and invasive abilities of GC cells (Figure 3E,F). Mechanistically, this anti‐metastatic effect was associated with the upregulated E‐cadherin and downregulated vimentin and N‐cadherin expression (Figure 3J). Furthermore, CDO1 overexpression significantly induced apoptosis in all three GC cell lines (Figure 3G). Three independent siRNA sequences targeting CDO1 were designed and transfected into gastric cancer cells (Figure S5C,D). The most effective siRNA (si‐CD01 #2) was selected for subsequent experiments. In contrast, CDO1 knockdown significantly promoted tumour progression, exhibiting effects opposite to those of CDO1 overexpression (Figure S5E–L). Besides, we performed CD8 immunohistochemistry on the same TMA cohort (*n* = 56). CDO1 showed a correlation with CD8+ T cell infiltration (Figure 3H). Furthermore, Western blotting revealed that CDO1 overexpression downregulated PD‐L1 and upregulated MHC class I in GC cells (Figure 3I). Consistently, the T‐cell infiltration score was positively associated with CDO1 expression (Figure S5M). Taken together, these results show that CDO1 suppresses tumour progression by impeding proliferation through cell cycle arrest and attenuating metastasis through EMT inhibition.

### 3.6 CDO1 suppresses proliferation and EMT via PI3K/AKT activation

**FIGURE 4:**
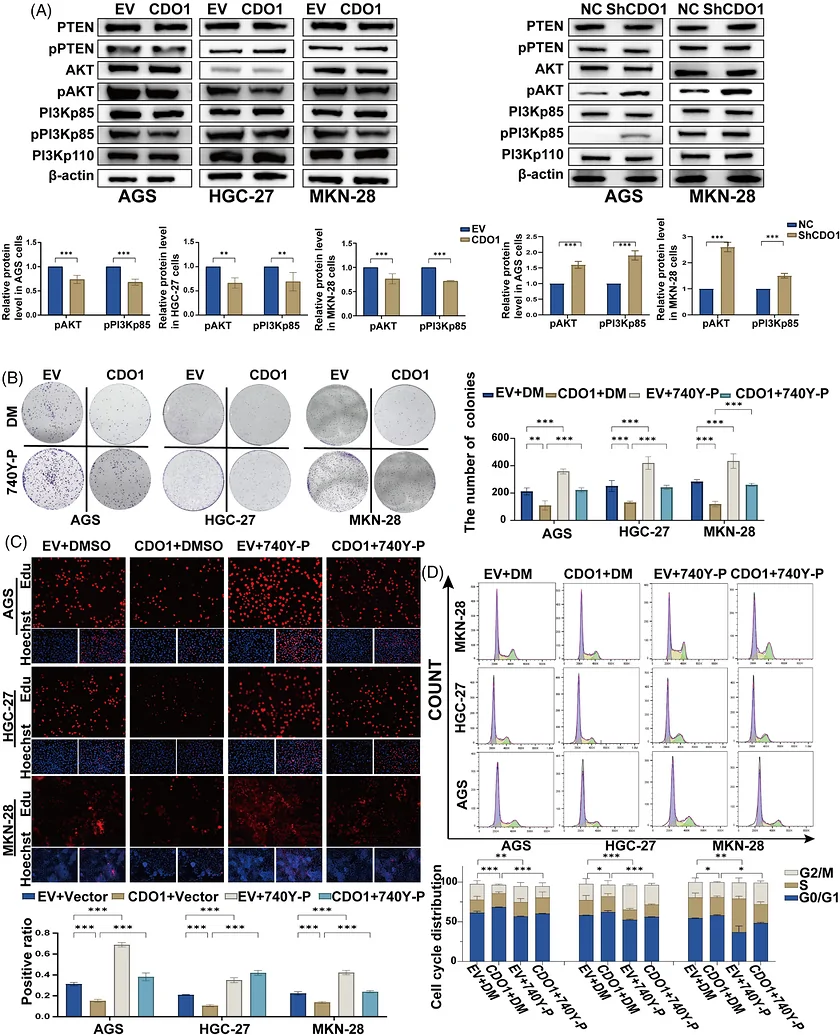
Pharmacological activation of PI3K by 740Y‐P reverses the oncostatic effects of CDO1 overexpression in GC cells. (A) Western blot analysis of PI3K/AKT pathway proteins in gastric cancer cells with CDO1 overexpression or knockdown. (B, C) Colony formation assay and EDU assay assessing the proliferative capacity of CDO1‐overexpressing cells treated with the PI3K activator 740Y‐P or vehicle control (DMSO) (scale bar = 50 µm). (D) Flow cytometric analysis of the cell cycle distribution rate in CDO1‐overexpressing cells following 740Y‐P treatment (*p < .05, **p < .01, ***p < .001, ns = not significant. Data are presented as mean ± SD; *n* = 3).

**FIGURE 5:**
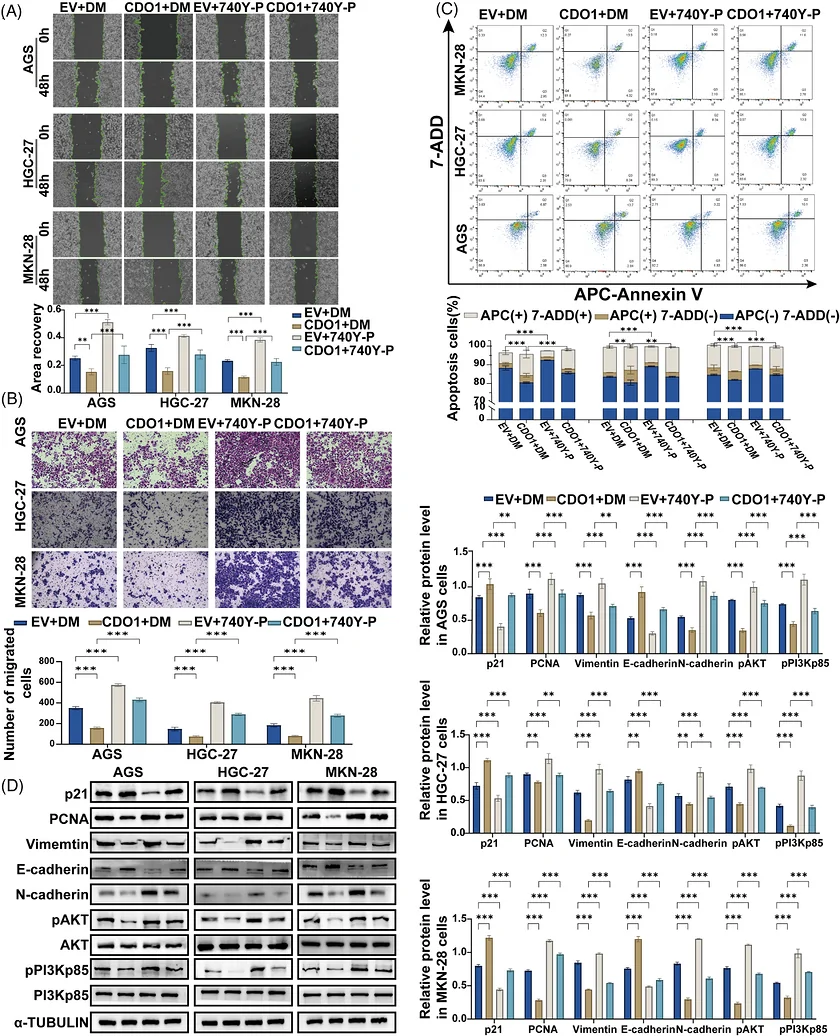
(A, B) Functional evaluation of metastatic potential via a wound‐healing assay and Matrigel invasion assay in CDO1‐overexpressing cells treated with 740Y‐P (scale bar = 50 µm). (C) Flow cytometric analysis of apoptosis in gastric cancer cells. (D) Western blot analysis of proliferation markers and EMT markers in CDO1‐OE cells treated with the PI3K activator 740Y‐P or vehicle (DMSO). 740Y‐P treatment reversed CDO1‐induced changes in these proteins (*p < .05, **p < .01, ***p < .001, ns = not significant. Data are presented as mean ± SD; *n* = 3).

To delineate the downstream pathways mediating the tumour‐suppressive effects of CDO1, we performed transcriptomic sequencing of control and CDO1‐overexpressing AGS cells. Differential expression analysis identified 758 DEGs. Enrichment analyses of these DEGs indicated that CDO1 overexpression was significantly linked to several cancer‐related pathways, including the PI3K‐AKT pathway, RAS pathway, DNA replication pathway, and cell cycle regulation pathway (Figure S6A,B). This observation was further validated through analysis of an independent TCGA‐STAD dataset. Stratifying patients by median CDO1 expression yielded 2187 DEGs whose enrichment profiles consistently highlighted the same set of pathways (Figure S6C–E). Therefore, we focused on validating PI3K‐AKT signalling, and western blotting confirmed that CDO1 upregulation specifically inhibited the phosphorylation of PI3Kp85 and AKT without affecting PI3Kp110 or PTEN levels, while CDO1 knockdown promoted PI3K and AKT phosphorylation (Figure 4A). To functionally establish this link, we stimulated CDO1‑OE cells with the PI3K activator 740Y‑P. 740Y‐P treatment effectively rescued the CDO1‐induced suppression of colony formation, EdU incorporation, and cell cycle progression (Figure 4B–D), and reversed the increased apoptosis observed in CDO1‐OE cells (Figure 5C). Furthermore, 740Y‐P restored the CDO1‐induced inhibition of migration and invasion, as shown by wound healing and Transwell assays (Figure 5A,B). Western blot analysis confirmed that 740Y‐P reversed the CDO1‐mediated changes in proliferation and EMT markers (Figure 5D). Collectively, our work demonstrates that the antiproliferative and antimetastatic functions of CDO1 are mediated by reduced phosphorylation of the PI3K/AKT pathway.

### 3.7 CDO1 suppresses THBS1 expression by inhibiting PI3K/AKT signalling

**FIGURE 6:**
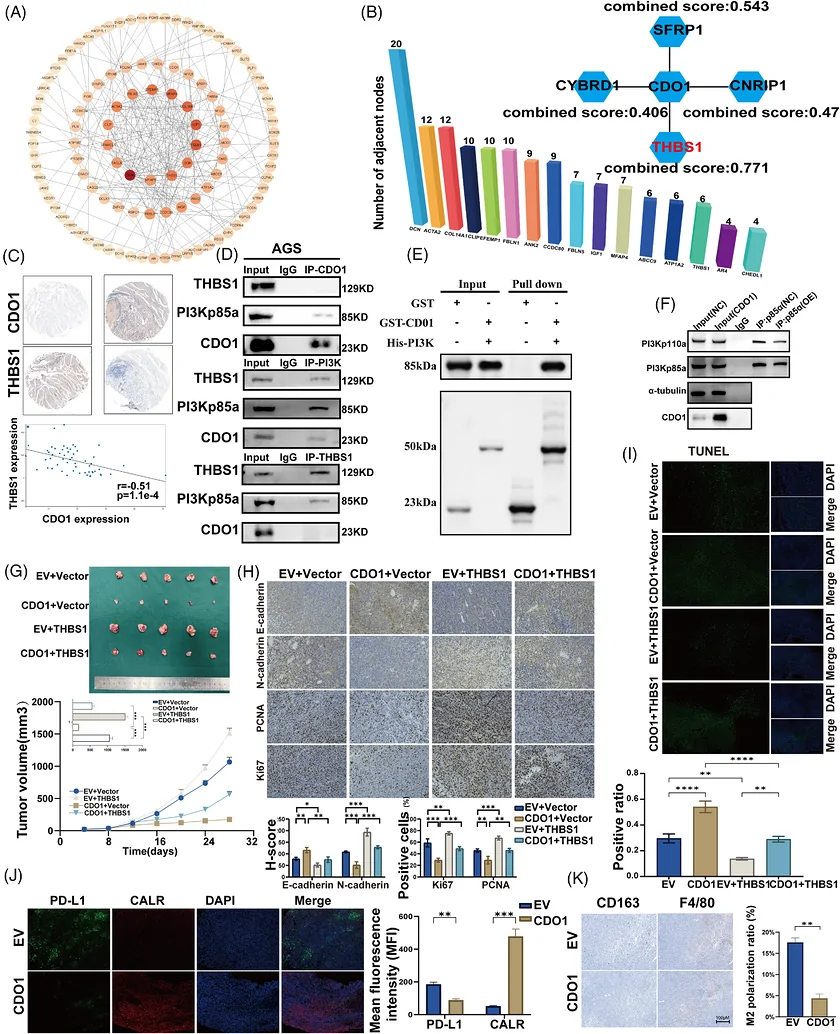
PI3K phosphorylation serves as a mediator through which CDO1 regulates THBS1 expression. (A) The PPI network for CDO1‐related genes was constructed and analyzed via the STRING database and Cytoscape 3.9 software. (B) Analysis of the STRING‐based PPI network reveals the interaction count per gene. Four genes were identified as direct neighbours of CDO1. (Cor: correlation coefficient; Score: STRING‐derived combined score). (C) CDO1 expression inversely correlates with THBS1 levels in the TMA cohort. (D) Co‐immunoprecipitation assays in AGS cells revealed mutual protein‒protein interactions among CDO1, PI3K (p85α), and THBS1. Input, whole‐cell lysate; IgG, negative control. (E) GST pull‐down assay demonstrating a direct interaction between CDO1 and the PI3K regulatory subunit p85α. (F) Co‐immunoprecipitation shows that CDO1 overexpression disrupts the p85α‐p110α interaction. (G) Representative images of tumours harvested at the study endpoint (day 28). BALB/c nude mice were engrafted with AGS cells stably expressing CDO1, THBS1, or empty vector. The tumours were excised for volumetric measurement (*V* = *L*×*W* ^2^/2). (H) Representative immunohistochemical images and quantitative analysis in tumour sections derived from the respective experimental groups (Scale bars = 30 µm). (I) Representative TUNEL staining images and quantification of the apoptotic index (Scale bars = 100 µm). (J) Multiplex IF staining of PD‐L1 (green), CALR (red), and DAPI (blue) in xenograft tumours from NC and CDO1‐OE groups (scale bar = 100 µm). (K) IHC staining of F4/80 (pan‐macrophage marker) and CD163 (M2 macrophage marker) (scale bar = 100 µm). (*p < .05, **p < .01, ***p < .001, ns  =  not significant. Data are presented as mean ± SD; *n* = 5).

To identify downstream mediators, we performed a correlation analysis (|*r*| ≥ .75, *p* < .05) in the TCGA‐STAD cohort and identified 187 transcripts markedly co‐expressed with CDO1. A protein‒protein interaction (PPI) network developed from these genes highlighted 16 hub nodes with the highest connectivity (Figure 6A). Among the four genes that directly interact with CDO1 in the network THBS1, CYBRD5, SFRP1, and CNRIP1 (Figure 6B), THBS1 was selected for further investigation on the basis of its high connectivity, strong correlation, and reported oncogenic roles.^34^, ^35^, ^36^ THBS1 upregulation in gastric cancer was validated by western blotting and qRT‒PCR in cell lines, as well as in the TCGA databases (Figure S7A–D). Immunohistochemical analysis of 30 paired clinical samples further corroborated these findings (Figure S7E). Consistently, univariate Cox regression revealed that elevated THBS1 levels contributed to poorer overall survival in pan‐cancer analyses (Figure S7F). In our in‐house gastric cancer TMA cohort, CDO1 protein expression was inversely correlated with THBS1 levels (Figure 6C), supporting the CDO1‐PI3K/AKT‐THBS1 regulatory axis in clinical specimens. Mechanistically, we found that THBS1 expression is modulated by the PI3K/AKT signalling. Treatment with the PI3K activator 740Y‐P markedly increased THBS1 protein levels (Figure S7G), confirming that PI3K activation positively modulates THBS1. CDO1 overexpression downregulated THBS1, whereas CDO1 knockdown upregulated THBS1 (Figure S7H). Co‐immunoprecipitation (Co‐IP) assays using AGS cell lysates revealed that endogenous CDO1 interacts with the PI3K regulatory subunit p85α (Figure 6D), suggesting their physical association. To confirm direct binding, GST pull‐down experiments were carried out using recombinant GST‐CD01 and His‐tagged p85α. GST‐CD01 efficiently pulled down His‐p85α, whereas GST alone did not (Figure 6E), confirming a direct physical interaction between CDO1 and p85α. We next investigated the functional consequence of this binding by examining the p85α‐p110α interaction. Co‐IP of p85α followed by immunoblotting for p110α showed that CDO1 overexpression markedly reduced the amount of p110α associated with p85α (Figure 6F). These results demonstrate that CDO1 directly binds p85α, thereby disrupting the PI3K heterodimer assembly and providing a mechanistic basis for attenuated AKT signalling. In summary, these data indicate that CDO1 suppresses THBS1 expression, likely through the inhibition of PI3K/AKT phosphorylation, positioning THBS1 as a critical downstream effector contributing to the tumour‐suppressive function of CDO1 in gastric cancer.

### 3.8 THBS1 facilitates CDO1‐driven proliferation and EMT in GC cells

To determine whether THBS1 is required for the tumour‐suppressive effects of CDO1, functional rescue was achieved by co‐overexpressing THBS1 in CDO1‑overexpressing GC cells, and THBS1 overexpression was verified by western blotting and qRT‒PCR (Figure S7J,K). THBS1 upregulation effectively reversed the CDO1‐mediated suppression of proliferation and apoptosis, and attenuated the inhibitory effects on cell migration, invasion, and EMT (Figure S8A–E; Figure S9A,B). These findings demonstrate that the growth‐inhibitory and metastasis‐suppressing functions of CDO1 are THBS1‑dependent in vitro. We validated this dependency in vivo via a subcutaneous xenograft model. Compared with control mice, mice implanted with AGS cells overexpressing CDO1 alone presented markedly suppressed tumour growth relative to controls. Co‐overexpression of THBS1 largely reversed this tumour‐suppressive phenotype (Figure 6G). Immunohistochemical analysis of tumour sections confirmed that CDO1 overexpression reduced proliferation (Ki‑67, PCNA) and inhibited EMT (increased E‑cadherin, decreased N‑cadherin), which was partially reversed by THBS1 co‐expression (Figure 6H). Consistently, the increased apoptosis observed in CDO1‑overexpressing tumours was attenuated upon THBS1 overexpression, as shown by the TUNEL assay (Figure 6I). Furthermore, in the CDO1‐OE xenograft model, multiplex IF and IHC analyses showed that CDO1 overexpression was associated with reduced PD‐L1 and increased CALR expression, indicating enhanced tumour‐intrinsic immunogenicity, as well as suppressed M2 macrophage polarization without altered total macrophage infiltration (Figure 6J,K). Our data indicate that CDO1 expression is correlated with enhanced tumour‐intrinsic immunogenicity and suppressed M2 macrophage polarization in vivo, and establish THBS1 as a key downstream effector of CDO1 tumour‐suppressive activity

### 3.9 Decitabine epigenetically restores CDO1 expression and exerts CDO1‐dependent anti‐tumour activity

**FIGURE 7:**
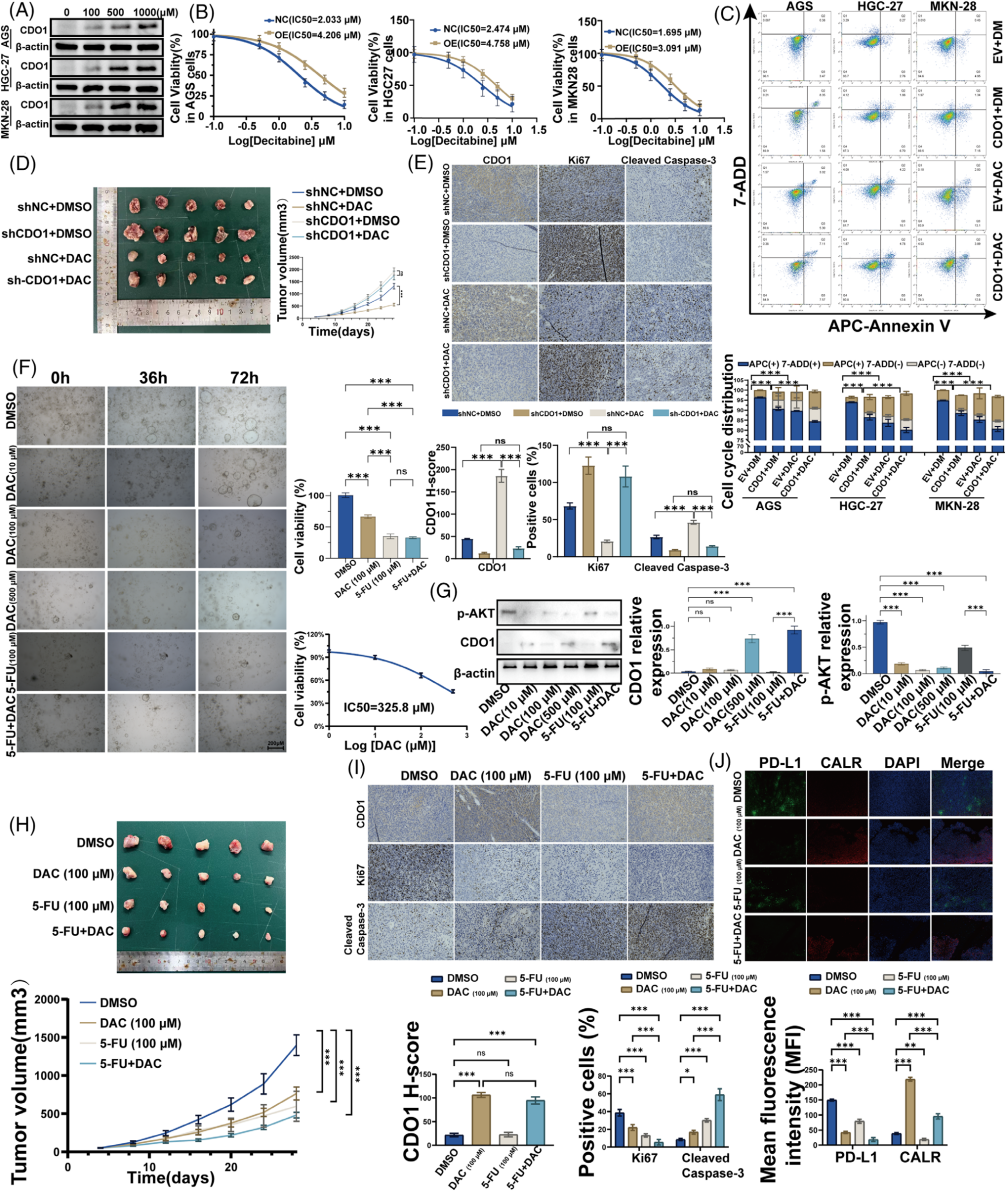
Decitabine epigenetically restores CDO1 expression and exerts CDO1‐dependent anti‐tumour activity in gastric cancer. (A) CDO1 protein levels in GC cells treated with decitabine (0, 100, 500, and 1000 µM, 48 h). (B) The IC_50_ of decitabine was determined via a CCK‐8 assay. (C) Apoptosis assays in cells treated with decitabine alone or in combination with CDO1 overexpression. (D) Tumour growth curves and final tumour weights in the 2×2 factorial xenograft model. AGS cells expressing control shRNA (sh‐NC) or CDO1‐targeting shRNA (sh‐CD01) were implanted into BALB/c nude mice and treated with vehicle or decitabine. (E) Representative immunohistochemical images and quantification of CDO1, Ki‐67, and Cleaved Caspase‐3 in tumour sections from the four groups. (F) Bright‐field images, dose‐response curve, and cell viability of patient‐derived gastric cancer organoids treated with the indicated concentrations of decitabine and 5‐FU, alone or in combination, for 72 h. Cell viability was measured by CellTiter‐Glo 3D assay (scale bar = 200 µm). (G)Western blot analyses of CDO1 and p‐AKT. (H) Tumour growth curves and final tumour weights of AGS xenografts in nude mice treated with decitabine, 5‐FU, or their combination. (I) Representative IHC images of CDO1, Ki‐67, and cleaved caspase‐3 in tumour tissues from the four treatment groups (scale bar = 30 µm). (J) Representative IF images of PD‐L1 and CALR (scale bar = 100 µm) (*p < .05, **p < .01, ***p < .001, ns =  not significant. Data are presented as mean ± SD; *n* = 5).

These findings prompted us to identify pharmacological agents capable of restoring CDO1 expression. Pharmacogenomic analysis using the GSCA platform identified 68 candidate drugs negatively correlated with our gene signature (*r* < −.15, *p* < .05). After integrating insights from the relevant literature, we narrowed the list to eight promising compounds for subsequent molecular docking studies against CDO1.^37^, ^38^, ^39^, ^40^ Among these, decitabine demonstrated the most favourable binding affinity (lowest docking energy) for CDO1. Exploratory molecular docking and molecular dynamics simulations were performed (Figure S10A,B). To investigate whether decitabine could upregulate the expression of CDO1 in GC, cells were exposed to decitabine at various concentrations (0, 100, 500, and 1000 µM) for 48 h. As shown by western blotting, decitabine treatment significantly and dose‐dependently upregulated the CDO1 protein levels in tumour cells (Figure 7A). The CCK‐8 assay demonstrated that CDO1 upregulation in GC cells resulted in an approximately twofold increase in the IC_50_ value for decitabine, which was accompanied by parallel dose‒response curves (Figure 7B). This pattern provides critical mechanistic insight into the characteristics of additive drug‒gene interactions. CDO1 exerts a constitutive tumour‐suppressive effect, lowering baseline cell viability. Consequently, decitabine must exert an additional proportional cytotoxic effect on this already suppressed population to achieve the same fractional inhibition (50%), thereby requiring a higher absolute concentration. This additive model strongly supports the conclusion that the antiproliferative action of decitabine converges with, and is independent of, the intrinsic pathway regulated by CDO1. We compared apoptosis across NC, CDO1‐OE, NC+Dec, and OE+Dec groups. The OE+Dec group exhibited the highest apoptotic rate, indicating that CDO1 overexpression sensitizes gastric cancer cells to decitabine‐induced apoptosis (Figure 7C–E). To directly test whether decitabine efficacy is CDO1‐dependent in vivo, we established a 2×2 factorial xenograft model using sh‐NC or sh‐CD01 AGS cells treated with vehicle or decitabine. Decitabine significantly suppressed tumour growth and induced apoptosis in control tumours, but these effects were largely abrogated by CDO1 knockdown (Figure 7D). Immunohistochemistry confirmed that decitabine‐induced restoration of CDO1, reduction of Ki‐67, and increase in Cleaved Caspase‐3 were markedly attenuated in sh‐CD01 tumours (Figure 7E). Thus, decitabine exerts CDO1‐dependent anti‐tumour activity in vivo.

### 3.10 Decitabine synergizes with 5‐FU to suppress gastric cancer growth in organoids and xenografts

To further validate these findings in a more clinically relevant system, we utilized patient‐derived gastric cancer organoids. Organoids were treated with decitabine at 10, 100, and 500 µM for 72 h, either alone or in combination with 5‐FU (100 µM). The CellTiter‐Glo 3D assay revealed a dose‐dependent reduction in organoid viability, yielding an IC_50_ of approximately 325.8 µM. Bright‐field imaging revealed that decitabine‐treated organoids exhibited growth arrest with intact morphology, whereas 5‐FU‐treated organoids displayed pronounced disintegration and cellular debris, reflecting their distinct cytotoxic versus cytostatic mechanisms (Figure 7F). Consistent with the results in 2D cell cultures, decitabine treatment significantly restored CDO1 mRNA and protein levels, leading to decreased phosphorylation of AKT (Figure 7G). We next evaluated the in vivo anti‐tumour activity of decitabine in a xenograft model. Animals were randomly distributed among four treatment arms: vehicle control, decitabine monotherapy, 5‐FU monotherapy, and decitabine plus 5‐FU combination. The combination of decitabine and 5‐FU produced the most pronounced tumour suppression, with markedly suppressed tumour volumes and weights compared with either monotherapy (Figure 7H). Immunohistochemical analysis confirmed that decitabine restored CDO1 expression, reduced proliferation, and induced apoptosis in tumour specimens, effects that were further enhanced by the addition of 5‐FU (Figure 7I). Immunofluorescence analysis further revealed that decitabine treatment downregulated PD‐L1 and upregulated CALR, consistent with enhanced tumour cell immunogenicity (Figure 7J). Collectively, these results demonstrate that decitabine suppresses gastric cancer growth by reactivating the CDO1 pathway, with its efficacy significantly potentiated by 5‐FU.

## 4 DISCUSSION

Gastric cancer continues to be a major contributor to global cancer mortality, with limited therapeutic targets and a persistently poor prognosis for advanced disease. Our findings establish CDO1 as a tumour suppressor in GC that is frequently silenced by promoter hypermethylation and whose low expression independently predicts poor prognosis.^10^, ^14^, ^41^, ^42^ Mechanistically, we demonstrate that CDO1 directly binds the PI3K regulatory subunit p85α, disrupting p85α–p110α heterodimerization to suppress AKT phosphorylation and THBS1 expression, thereby inhibiting tumour cell proliferation, migration, and EMT.^43^, ^44^, ^45^ This CDO1–PI3K/AKT–THBS1 axis provides a molecular framework explaining the inverse correlation between CDO1 and THBS1 in clinical specimens and rationalizes the dual tumour‐suppressive effects of CDO1 on both proliferative and metastatic programs.

A particularly intriguing finding of this study is that CDO1, a metabolic enzyme canonically defined by its cysteine dioxygenase activity, appears to suppress oncogenic signalling through a direct protein–protein interaction with p85α. This adds CDO1 to a growing list of metabolic enzymes with non‐canonical moonlighting roles, such as PKM2, which translocates to the nucleus to phosphorylate histone H3 independent of its glycolytic activity, and PGK1, which serves as a protein kinase to regulate glycolysis and the TCA cycle. However, we acknowledge that the current data cannot distinguish whether CDO1‐mediated PI3K/AKT inhibition requires its enzymatic activity or is solely dependent on this protein‐scaffold mechanism. It remains possible that CDO1's catalytic function—oxidizing cysteine to produce taurine—contributes to tumour suppression through metabolic reprogramming or redox regulation, while the p85α interaction serves an auxiliary or context‐dependent role. Alternatively, CDO1 may have evolved a scaffolding function entirely separable from its enzymatic activity. Resolution of this question will require systematic structure‐function studies employing enzyme‐dead CDO1 mutants (e.g., Fe^2^^+^‐coordinating residue mutations) and p85α binding‐deficient CDO1 mutants in functional rescue experiments. Such investigations will be essential to elucidate whether CDO1's tumour‐suppressive function is catalysis‐dependent or relies on direct disruption of the PI3K heterodimer, and may inform the design of CDO1‐mimetic peptides or small molecules that bypass the need for enzymatic restoration.

Beyond its cell‐intrinsic tumour‐suppressive role, our bioinformatic and tissue microarray analyses reveal a significant association between CDO1 expression and an immune‐inflamed tumour microenvironment. High CDO1 expression was associated with enhanced CD8+ T cell infiltration in clinical TMA specimens, and with elevated expression of immune checkpoint molecules including BTLA and PD‐L1.^46^, ^47^, ^48^ In xenograft models, CDO1 overexpression was associated with reduced PD‐L1 and increased CALR expression, indicating enhanced tumour‐intrinsic immunogenicity, as well as suppressed M2 macrophage polarization, suggesting a potential impact on the innate immune compartment. Notably, PI3K/AKT signalling has been implicated in regulating PD‐L1 expression and immune evasion; thus, CDO1‐mediated PI3K/AKT inhibition may coordinately suppress tumour cell proliferation while enhancing tumour‐intrinsic immunogenicity. However, we emphasize that the current evidence for CDO1's immunomodulatory role remains largely correlative. Critically, our in vivo functional experiments were performed in BALB/c nude mice, which lack mature T cells and therefore cannot recapitulate adaptive immune responses. Consequently, all immune‐related observations from xenograft models must be interpreted strictly within the context of tumour‐intrinsic immunogenicity (PD‐L1 downregulation, CALR upregulation) and innate immunity (macrophage polarization). The association between CDO1 expression and CD8+ T cell infiltration is supported exclusively by clinical TMA data and bioinformatic analyses, not by functional evidence from immunocompetent models. Future studies employing syngeneic mouse models or humanized mice will be necessary to determine whether CDO1 directly drives T cell‐mediated anti‐tumour immunity or is a bystander marker of an immunogenic tumour context.

Therapeutically, we demonstrate that decitabine, a clinically approved DNMT inhibitor, epigenetically restores CDO1 expression in gastric cancer cells and patient‐derived organoids.^49^, ^50^, ^51^, ^52^ Importantly, our CDO1‐knockdown xenograft experiment provides direct in vivo evidence that the anti‐tumour efficacy of decitabine is critically dependent on CDO1 reactivation, as CDO1 deficiency largely abrogated decitabine‐mediated tumour suppression, Ki‐67 reduction, and Cleaved Caspase‐3 induction. This CDO1‐dependent mechanism offers a rationale for biomarker‐guided patient stratification: gastric cancer patients with low tumoral CDO1 expression or CDO1 promoter hypermethylation may derive the greatest clinical benefit from decitabine‐based epigenetic therapy. Furthermore, the synergy between decitabine and 5‐fluorouracil observed in our organoid and xenograft models suggests that combining epigenetic reactivation of tumour suppressors with standard chemotherapy warrants further preclinical investigation, particularly since 5‐FU‐based regimens remain the backbone of gastric cancer treatment. In xenograft models, decitabine treatment reduced PD‐L1 and increased CALR expression, suggesting enhanced tumour‐intrinsic immunogenicity. We acknowledge that decitabine‐mediated demethylation is not gene‐specific—it simultaneously reactivates multiple epigenetically silenced loci. While our data identify CDO1 as a primary effector, the contribution of other demethylated genes to the overall anti‐tumour phenotype cannot be fully excluded. Future studies integrating genome‐wide methylation profiling with CDO1‐knockout models will help delineate the CDO1‐specific versus pleiotropic effects of decitabine.

In summary, this study establishes CDO1 as both a tumour suppressor and a prognostic indicator in GC, reveals a CDO1–PI3K/AKT–THBS1 signalling axis, and supports the epigenetic reactivation of CDO1 by decitabine as a translatable therapeutic strategy.^39^, ^53^, ^54^, ^55^ The direct CDO1–p85α interaction provides a structural basis for PI3K/AKT inhibition, while the CDO1‐dependent anti‐tumour activity of decitabine provides a mechanistic rationale for clinical translation. Future studies integrating the enzymatic, scaffolding, and immunological functions of CDO1 will be essential to fully harness its therapeutic potential in gastric cancer and potentially other CDO1‐silenced malignancies.

## 5 CONCLUSION

In conclusion, this study establishes CDO1 as a prognostic biomarker and tumour suppressor in gastric cancer that operates through a CDO1–PI3K/AKT–THBS1 signalling axis. Multi‐omics analysis and clinical validation identified CDO1 as frequently downregulated by promoter methylation, with its low expression independently predicting poor prognosis. Crucially, CDO1 directly binds the PI3K regulatory subunit p85α, disrupting p85α–p110α heterodimerization to suppress AKT phosphorylation and THBS1 expression, thereby inhibiting tumour cell proliferation, migration, and EMT. Significantly, decitabine epigenetically restores CDO1 expression, and its in vivo anti‐tumour efficacy is critically dependent on CDO1 reactivation, synergizing with 5‐fluorouracil in patient‐derived organoids and xenograft models, highlighting a translatable epigenetic strategy for gastric cancer treatment.

## AUTHOR CONTRIBUTIONS

Y.W., Y.A., and Y.Y. designed and oversaw this project. The experimental work was carried out by F.W. and R.Z. Figure preparation and data visualization were performed by J.C. and J.W. The data analysis and interpretation were led by F.W., who also drafted the manuscript. The final manuscript was reviewed and approved by all the authors.

## CONFLICT OF INTEREST STATEMENT

The authors declare no conflict of interest.

## ETHICS APPROVAL AND CONSENT TO PARTICIPATE

Animal Experiments: This study involving animals was performed following ethical review and oversight by the Animal Ethics Committee of Shanghai General Hospital (approval number: 2025AW030). All procedures strictly adhered to the 3R principles and animal welfare standards, with specific measures implemented to alleviate pain and distress and to employ humane endpoints where necessary.

## HUMAN PARTICIPANTS

This study was conducted in accordance with the principles of the Declaration of Helsinki and was approved by the Medical Ethics Committee of Shanghai General Hospital (Approval Number: 2024SQ258). Written informed consent was obtained from all participants or their legal guardians before their enrolment in the study.

## Supporting information



Supplementary Information

Supplementary Information

Supplementary Information

Supplementary Information

Supplementary Information

Supplementary Information

Supplementary Information

Supplementary Information

Supplementary Information

Supplementary Information

Supplementary Information

Supplementary Information

Supplementary Information

## Data Availability

The RNA‐seq data generated in this study have been deposited in the Gene Expression Omnibus (GEO) under accession number GSE339180. All other data supporting the findings of this study are available within the article and its Supporting Information Materials. Publicly available datasets analyzed in this study can be accessed from TCGA, GEO, GTEx, and CPTAC as described in the Methods section. Any additional data that are not publicly available owing to privacy or ethical restrictions can be made available from the corresponding author upon reasonable request.
